# Reply: Methodological and Clinical Concerns in the Study on Postpartum Hypertension Management

**DOI:** 10.1016/j.jacadv.2025.101853

**Published:** 2025-05-31

**Authors:** Emily B. Rosenfeld, Rachel Lee, Deepika Sagaram, Todd Rosen, Cande V. Ananth

**Affiliations:** Rutgers Robert Wood Johnson Medical School, New Brunswick, New Jersey, USA

We thank Dr Tanamoto and colleagues for their interesting questions regarding our paper.^1^ They raise several questions, which we clarify below.

The writers’ first question is the study's clinical confounders in the propensity score match and inquire about mutlifetal gestation, smoking, alcohol use and highest blood pressure. They fail to note that multifetal gestation is matched as described in the statistical analysis section of the manuscript. Similarly, as indicated in our paper, we did consider additional variables, including smoking and alcohol (smoking n = 29, alcohol n = 7). Due to small numbers, consideration of these variables in the propensity score model resulted in an imprecise prediction, as acknowledged in the manuscript. As to why we did not consider including the highest blood pressure, since blood pressure is one of the primary endpoints, including this variable would reduce the variability in blood pressure measurements between the retrospective and prospective cohorts in the MOPP trial.

Second, the writers question the methodological approach to the data analyzed. Since missing data and covariates were imputed 15 times based on the multiple imputation methodology, calculating the standardized mean difference was cumbersome. However, we estimated the standardized mean differences before and after the propensity score match. For brevity, the range of the standardized mean difference before matching across all covariates is −0.17, 0.68; in the propensity score matched cohort, the corresponding range was −0.09, 0.17.

At the request of the authors, we re-ran the analyses to estimate the risk differences in the matched cohort, taking the matching into consideration. The results show identical risk difference estimates but with conservative CIs (which are tighter after accounting for the matching compared to what is reported in the manuscript).

As previously described in the statistical analysis section of the paper, doubly robust estimation was implemented to minimize the outcome's confounding bias. Since propensity score analyses minimize the exposure's confounding bias, the propensity score method does not address the outcome's confounding. Therefore, a doubly robust estimation method was necessary, and we implemented this method throughout the analysis for outcomes in the propensity score-matched cohort.

In their response, the authors misconstrue overadjustment and inappropriate adjustment. Overadjustment refers to scenarios where an intermediate variable on the causal pathway (or its descending proxy) is adjusted. Doing so will either increase the net bias or decrease the estimate's precision without affecting the bias.^2^ In contrast, unnecessary adjustments will not bias the effect estimate but may reduce its precision.^3^^,^^4^

When the authors inquire about the number of controls in the propensity score match, they raise an interesting point worthy of consideration. To address the potential for any bias based on the number of matches, we additionally replicated all the analyses by applying the inverse probability weighting method utilizing all subjects (Supplemental Table 2).^1^ The results of these analyses were similar to those reported as the primary outcome, suggesting that bias is unlikely in the reported associations.

The author’s letter does not specify their clinical concerns, but we hope this response clarifies the methodology of the MOPP trial.
